# Vapor-Phase Polymerized Poly(3,4-Ethylenedioxythiophene) on a Nickel Nanowire Array Film: Aqueous Symmetrical Pseudocapacitors with Superior Performance

**DOI:** 10.1371/journal.pone.0166529

**Published:** 2016-11-18

**Authors:** Qisen Xie, Yang Xu, Zhipeng Wang, Chao Xu, Peichao Zou, Ziyin Lin, Chenjie Xu, Cheng Yang, Feiyu Kang, Ching-Ping Wong

**Affiliations:** 1 Division of Energy and Environment, Graduate School at Shenzhen, Tsinghua University, Xili University Town, Nanshan District, Shenzhen City, Guangdong Province, China; 2 School of Materials Science and Engineering, Georgia Institute of Technology, Atlanta, Georgia, United States of America; 3 School of Chemical & Biomedical Engineering, Nanyang Technological University, Singapore, Singapore; University of Florida, UNITED STATES

## Abstract

Three-dimensional (3D) nanometal scaffolds have gained considerable attention recently because of their promising application in high-performance supercapacitors compared with plain metal foils. Here, a highly oriented nickel (Ni) nanowire array (NNA) film was prepared via a simple magnetic-field-driven aqueous solution deposition process and then used as the electrode scaffold for the vapor-phase polymerization of 3,4-ethylenedioxythiophene (EDOT). Benefiting from the unique 3D open porous structure of the NNA that provided a highly conductive and oriented backbone for facile electron transfer and fast ion diffusion, the as-obtained poly(3,4-ethylenedioxythiophene) (PEDOT) exhibited an ultra-long cycle life (95.7% retention of specific capacitance after 20 000 charge/discharge cycles at 5 A/g) and superior capacitive performance. Furthermore, two electrodes were fabricated into an aqueous symmetric supercapacitor, which delivered a high energy density (30.38 Wh/kg at 529.49 W/kg) and superior long-term cycle ability (13.8% loss of capacity after 20 000 cycles). Based on these results, the vapor-phase polymerization of EDOT on metal nanowire array current collectors has great potential for use in supercapacitors with enhanced performance.

## Introduction

Supercapacitors are a type of energy storage device with great promise due to their fast dynamic response (i.e., high rate capability), high power density, and exceptionally long cycle life compared with conventional batteries [1–4]. supercapacitors have been employed in a wide variety of applications ranging from portable electronic equipment to hybrid electric vehicles, backup power sources and large-scale power grid management [3,5–7]. Based on their energy storage mechanism, supercapacitors can be divided into electrochemical double layer capacitors (EDLCs) and pseudo-capacitors. EDLC electrode materials, e.g., carbon nanotubes and graphene, have been intensively studied due to their high electrical conductivity and cycling capability [4,8]. However, as a conventional electrode material, the hydrophobic nature of highly graphitized carbon is disadvantageous to the solution-phase ion infiltration, thus leading to inferior capacitance [9]. Pseudo-capacitors, such as transition metal oxides and conducting polymers, can provide superior specific capacitance (10 to 100 times higher than carbon materials) through the fast redox reactions at the electrolyte/electrode interface [4,10,11]. Conducting polymers are especially attractive candidates due to their excellent structural stability, reaction reversibility, and superior electrical conductivity [12]. Among the various conducting polymers, poly(3,4-ethylenedioxythiophene) (PEDOT) possesses excellent environmental stability, a narrow bandgap (e.g., ca. 1.6 eV), a low oxidation potential, a wide potential window for both positive (p-) and negative (n-) doping, environmentally benign characteristics, high electrical conductivity and good optical transparency as a result of its linear structure without the appearance of *α*,*β*-mislinking [13], which makes it an ideal prototypical material for electrochromics [14], solar cells [15], fuel cells [16], and supercapacitors [17].

The electrochemical charge storage of PEDOT is related to the surface doping and de-doping process, which largely depends on the intrinsic conductivity and surface area of the PEDOT-based electrode [17]. Numerous efforts have been made to enhance the capacitive performance of PEDOT-based electrodes [17,18]. For instance, Anothumakkool et al. fabricated cellulose-based PEDOT conductive paper as both a current collector and freestanding electrode via an interfacial polymerization reaction [17]. D’Arcy et al. developed a PEDOT electrode with excellent intrinsic conductivity via vapor-phase polymerization for use in a high-performance supercapacitor application [18]. However, the cycling stability of PEDOT-based electrodes has remained poor mainly due to the large volumetric swelling and shrinking during repeated doping/de-doping (charge/discharge) [19].

Along with the above considerations, many efforts have focused on improving the electrochemical stability of conducting polymers, such as the development of novel nanostructured material systems [18] and advanced multifunctional nanocomposites [20] and the selection of electrolytes with excellent ionic conductivity and electrochemical stability [21]. Among these efforts, the use of rationally designed three-dimensional (3D) hierarchical structures is a promising approach. These 3D nanostructured electrodes not only facilitate charge transfer and mass diffusion through an improved level of active material loading but also accommodate large-volume expansions to release stress [22,23]. In contrast to the available carbon-based nanocomposites, conductive metallic nano-architectures would be more suitable as 3D electrode scaffolds due to several advantages, e.g., facile fabrication of pseudocapacitive material systems resulting from their hydrophilicity, superior electrical conductivity, and mechanical robustness [24]. For example, after the facile coating of MnO_2_ on electronically conductive copper (Cu) superstructures, the resulting MnO2/Cu composite exhibited a high specific capacitance of 1024 F/g at 1.5 A/g and good capacity retention of 96% after 2000 cycles at 1.5 A/g [25]. Su et al. developed an amorphous Ni(OH)2@3D nickel (Ni) core-shell complex electrode that had a large specific capacitance (2868 F/g at 1 mV/s) and good cycling stability (3% loss after 1000 cycles at 100 mV/s) compared to a conventional Ni(OH)_2_ electrode [26]. Additionally, 3D Ni@MnO_2_ and 3D Ni@polypyrrole (PPy) composites on supercapacitor electrodes with a greatly enhanced capacitive performance were developed that were superior to the pure active materials [27,28].

In this study, a simple, direct, and scalable vapor-phase polymerization approach was used for the in situ preparation of PEDOT on a vertically aligned Ni nanowire array (NNA). Due to the unique morphological and structural characteristics, the NNA provided high-quality electron and ion pathways for the PEDOT active material, and the surface convexity helped release the stress generated during the cycling process. Thus, the NNA@PEDOT hierarchically structured electrode is very beneficial for delivering superior capacitive and cycle-life performances. The NNA@PEDOT hybrid films were further assembled into a symmetric supercapacitor, for which superior cycling performance and specific energy density were characterized and found to be comparable to some of the best results ever reported for all polymer-based supercapacitors. This work provides elaborate insights in addressing critical problems with conductive polymer-based electrochemical energy storage technology.

## Materials and Methods

### Materials

Iron(III) p-toluenesulfonate (Fe(PTS)_3_) (Sigma-Aldrich) was dried under vacuum before use. 3,4-Ethylenedioxythiophene (EDOT, Tokyo Chemical Industry) was distilled under reduced pressure prior to use. All other chemicals were used without further purification.

### Fabrication of NNAs

The NNA samples were fabricated via a two-step chemical deposition process, which was described in our recent work [23]. Briefly, the solutions were prepared with analytical-grade chemicals dissolved in deionized (DI) water. A commercial Ti foil (99.99%, thickness ca. 40 μm) was used as the substrate film. Firstly, a thin layer of zinc (Zn) was electrodeposited onto the Ti foil in a solution containing 1.0 M ZnSO_4_ and 3.0 M KCl at a current density of 2 mA/cm^2^ for 60 s. The film was washed with DI water and dried in an oven at 60°C for 2 h, followed by immersion in a Pd plating bath containing 0.023 M PdCl_2_, 0.4 vol% HCl, 30 vol% ammonia, and 5 mM hydrazine at 60°C for 30 min. After the active Pd layer was deposited, the sample was thoroughly washed with DI water and dried in air.

The NNA was deposited onto this film in situ using a modified method based on Kawamori et al. [22]. Briefly, 50 mL of an aqueous solution (defined as solution A) containing 0.10 M NiCl_2_, 37.5 mM sodium citrate (Na_3_C_6_H_5_O_7_) and 0.20 mM H_2_PtCl_6_ was prepared. The same amount of aqueous solution (50 mL) containing 8.5 vol% N_2_H_4_ was also prepared (solution B). Afterwards, the aforementioned Ti foil was placed inside a beaker vertical to the magnetic field direction from a piece of neodymium iron boron magnet that was fixed outside. These two items were placed as close as possible to each other. Prior to the reaction, the pH of solutions A and B was adjusted to 12.5 with 6 M aqueous KOH at room temperature, as measured by a pH meter (HORIBA, F-71). Solutions A and B were stored at 80°C for ~50 min and then mixed together into the aforementioned beaker, which was then placed in an 80°C water bath. After reaction for 1 h, the NNA with a height of up to 1 mm could be deposited onto the active Pd layer. Finally, the NNA samples were washed with DI water and ethanol three times and then dried in a desiccator at 60°C for 2 h.

### Vapor-phase Polymerization of EDOT

An acetonitrile solution of 7 wt% Fe(PTS)_3_ was used as the oxidant to initiate the polymerization reaction. Compared with other Fe(III) salts, Fe(PTS)_3_ is less easy to crystallize and can thus form a uniform thin film that improves the quality of the PEDOT film. The aforementioned NNA samples were employed as the current collector and mechanical support for the electrode materials. The NNA (1×1 cm^2^) sample was dipped into the Fe(PTS)_3_ solution for 1 minute and then transferred into an oven at 70°C to allow evaporation of the solvent and enable the formation of a uniform coating of oxidant on this framework. The NNA was then placed in a sealed glass cell filled with 0.05 mL of EDOT and heated to 90°C for 2 h for the vapor-phase polymerization of the monomer. Subsequently, the NNA@PEDOT electrode was washed with ethanol and DI water three times to remove the residual iron salt and then dried at 60°C for 3 h. For clear presentation, this procedure was denoted as 1 vapor-phase polymerization cycle. The mass loading density was calculated based on the mass change before and after the preparation of the PEDOT electrode material. PEDOT was also deposited on Ni foam (NF) to construct NF@PEDOT for comparison, for which the deposition conditions were the same as those for the NNA@PEDOT electrode.

### Electrochemical polymerization of EDOT

The NNA@PEDOT electrode was fabricated by an electrochemical polymerization method for a control study. PEDOT was prepared in a solution containing 0.01 M EDOT, 0.01 M NaClO_4_, and 0.01 M NaPSS (Mw = 70 000) at a constant potential of 1.2 V. The mass loading was adjusted to be close to that of NNA@PEDOT via the vapor-phase polymerization process (ca. 0.22 mg/cm^2^).

#### Characterizations

The morphologies of the as-prepared samples were analyzed by field emission scanning electron microscopy (FE-SEM, Hitachi S4800, Japan) and transmission electron microscopy (TEM, JEOL-2100F). Fourier transform infrared (FTIR) spectra were obtained on a Thermo Scientific Nicolet iS 50 with the samples pressed in KBr pellets. The electrochemical properties of the as-prepared samples were investigated by cyclic voltammetry (CV), galvanic charge/discharge (GCD) measurements, and electrochemical impedance spectroscopy (EIS) on a CHI 760D electrochemical workstation (Chenhua, Shanghai).

### Electrochemical measurements

The electrochemical properties of the as-prepared electrodes were evaluated on an electrochemical station (VMP3, BioLogic, France). The CV and GCD measurements were performed in a three-electrode system (active materials as the working electrode, a platinum foil as the counter electrode, and a saturated calomel electrode as the reference electrode) with 0.5 M aqueous Na_2_SO_4_ as the electrolyte. The cycle-life tests were performed from the GCD measurements. EIS was performed over a frequency range from 100 kHz to 0.01 Hz at amplitude of 5 mV.

## Results and Discussion

**Fig 1:**
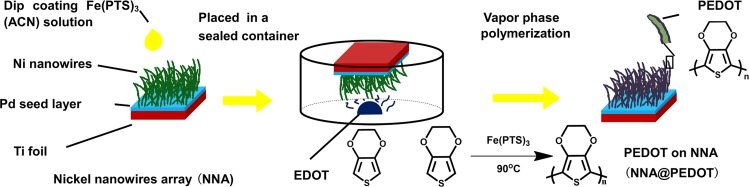
Schematic illustration of the preparation of the NNA@PEDOT electrode.

The preparation of NNA@PEDOT via vapor-phase polymerization is schematically demonstrated in Fig 1. The NNA was prepared through a modified magnetic-field-driven selective deposition growth process, which enabled the metallurgical bonding of NNA to the Pd nanoparticle interlayer grown on the Ti substrate. In this way, the NNA showed better scalability and a higher aspect ratio compared with other available technologies [22,26,27,29]. Due to the large magnetic moment of Pd nanoparticles, the adhesion between Ni and Ti was greatly strengthened [23]. The vertically aligned NNA presented unique electrical conductivity and mechanical properties [23]. Unlike the undesirable *α*,*β- or β*,*β’*-coupling present in PPy or polythiophene, the blocking of the *β*-position in PEDOT could lead to a more regionally regular structure with a coplanar heterocyclic backbone and highly oxygenic pendant group [30]. Compared to the in situ chemical or electrochemical polymerization methods, vapor-phase polymerization is more likely to generate PEDOT films with higher crystallinity and conductivity [18]. Furthermore, the substrates conventionally employed in vapor-phase polymerization usually have poor conductivities, and an additional current collector is essential [31]. The highly hydrophilic and conductive NNA developed in the present study can provide a 3D open porous scaffold for the facile deposition of PEDOT via the vapor-phase polymerization method. The color change of the NNA from black to dark blue implies the formation of PEDOT on the surface of the NNA film.

**Fig 2:**
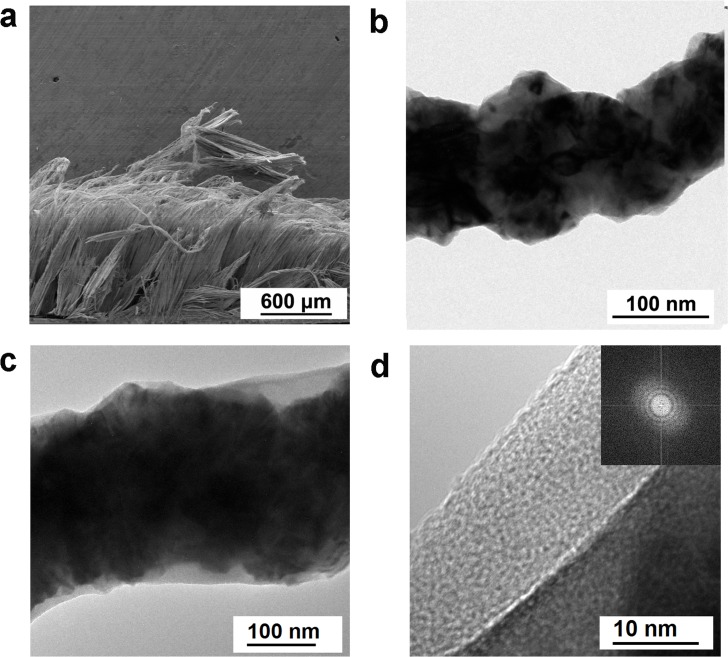
(a) SEM image of a cross-section of the NNA; (b) TEM image of a single Ni nanowire exfoliated from the NNA; (c) and (d) TEM images of a single piece of Ni nanowire covered by PEDOT via vapor-phase polymerization.

As shown in Fig 2A and 2B, the Ni nanowires with a high aspect ratio (diameter of 120~170 nm and length of ca. 1 mm, ratio respect of over 8000) were vertically aligned and densely packed on the substrate [23,32]; This featured textural property of NNA with high ratio respect could offer more sites for PEDOT coating and the coating would be thinner under the same mass loading compared to that with lower ratio aspect. The nanowire structure of NNA can improve the ion and electron transport between the current collector and active materials. In addition, Irregular surface of Ni nanowires with plenty of convexity can be observed from Fig 2B and Figure B in S1 File, and the surface of the as-obtained NNA @PEDOT electrode was smooth, as shown in Figure B in S1 File, which benefit from the PEDOT shell fabricated by vapor-phase polymerization. The uniformly distributed S, C, and O elements detected via energy dispersive spectroscopy mapping (Figure A in S1 File) further demonstrated the existence of the epitaxially and conformally coated PEDOT on the Ni nanowires after 3 cycles of the vapor-phase polymerization process. As displayed in Fig 2C and 2D, NNA @PEDOT presented a hierarchical structure and the average thickness of PEDOT coating was confirmed to be 12 nm. Besides, the amorphous PEDOT layer was further verified by fast Fourier transform analysis (Fig 2D, inset).

**Fig 3:**
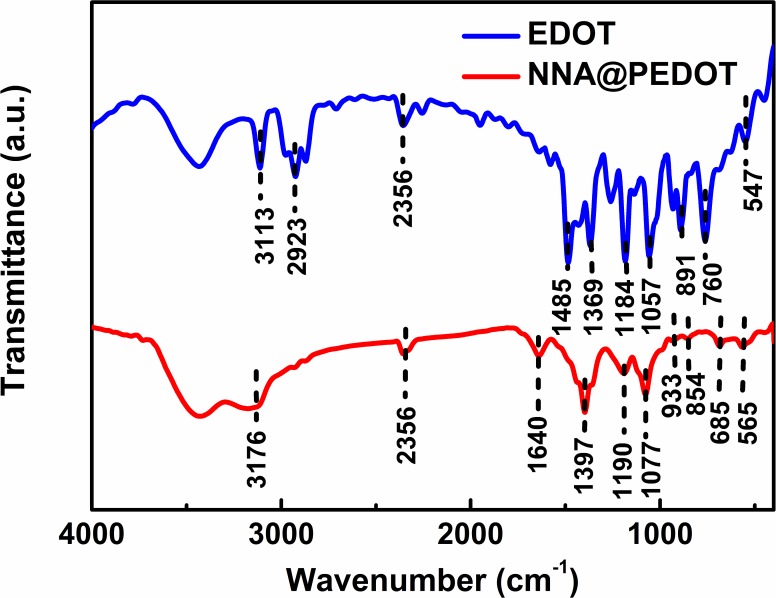
FTIR spectra of EDOT and NNA@PEDOT.

The vapor-phase polymerization of EDOT proceeded successfully in the sealed chamber, as demonstrated by the FTIR results in Fig 3. The strong bands at 1184 and 891 cm^−1^ for EDOT were attributed to ═C─H in-plane and out-of-plane deformation vibrations, respectively. Moreover, the band at 3113 cm^−1^ for EDOT was ascribed to ═C─H vibrations of the thiophene ring. However, the disappearance of these peaks in the polymer revealed the successful formation of PEDOT mainly via *α*,*α*’-linking [33]. The broad bands at 1400~1500 cm^−1^ for PEDOT were assigned to the stretching mode of the aromatic C═C band. The vibrational mode from the C─C bond in the thiophene ring was found at 1397 cm^−1^. In addition, the peaks at 1190 cm^−1^ and 1077 cm^−1^ were assigned to the stretching modes of the ethylenedioxy group. The bands at 933, 854, and 685 cm^−1^ were due to vibrations of the C─S bond in the thiophene ring. The relatively broad and sharp bands at 3428 cm^−1^ and 1640 cm^−1^ were assigned to the stretching and bending vibrations of H_2_O molecules, respectively [34].

**Fig 4:**
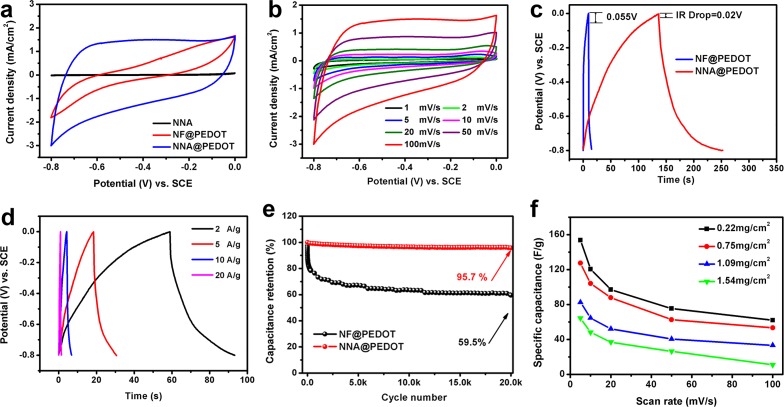
(a) CV curves of bare NNA, NNA@PEDOT, and NF@PEDOT at 100 mV/s; (b) CV curves of NNA@PEDOT at different scan rates; (c) IR drops of NNA@PEDOT and NF@PEDOT; (d) GCD curves of NNA@PEDOT at different current densities; (e) cyclic performance of NNA@PEDOT and NF@PEDOT at 5 A/g; (f) rate performance of NNA@PEDOT with different mass loadings at 5, 10, 20, 50, and 100 mV/s.

Fig 4A displays the CV curves of the NNA@PEDOT and NF@PEDOT samples with the same mass loading of 0.22 mg/cm^2^ in a potential window of -0.8 V to 0 V at 100 mV/s. The capacitance contribution of NNA was almost negligible when comparing the current density of pristine NNA with that of NNA@PEDOT, indicating that the function of NNA was similar to that of NF (as the current collector) [27]. However, the specific capacitance of NNA@PEDOT was much higher than that of NF@PEDOT, which was confirmed by both GCD and EIS studies (62.14 F/g and 17.42 F/g, respectively, at 100 mV/s). As shown in Fig 4C, the IR drop of NNA@PEDOT (0.02 V) was lower than that of NF@PEDOT (0.05 V), which led to their different capacitances. As observed in Figures C and I in S1 File, according to the EIS analysis of the NNA@PEDOT electrode that had been fitted into an equivalent electrical circuit using the nonlinear least-squares method, the electrolyte (*Re*) and charge transfer (*Rct*) resistances of NNA@PEDOT were 6.53 Ω and 4.26 Ω, respectively, smaller than those of NF@PEDOT (7.36/37.79 Ω of *Re/Rct*). This result demonstrates that the ion diffusion in the NNA was as fast as that in the NF, whereas the electron transfer in the NNA was much more efficient than that in the NF mainly because of the excellent electrical conductivity of the NNA as the current collector.

Notably, NNA@PEDOT from vapor-phase polymerization delivered much higher specific capacitance than NNA@PEDOT prepared by the electrochemical polymerization method (62.14 F/g and 25.86 F/g at 100 mV/s, respectively, Figure D in S1 File), implying that the vapor-phase polymerization was more suitable for the preparation of PEDOT in supercapacitors.

**Fig 5:**
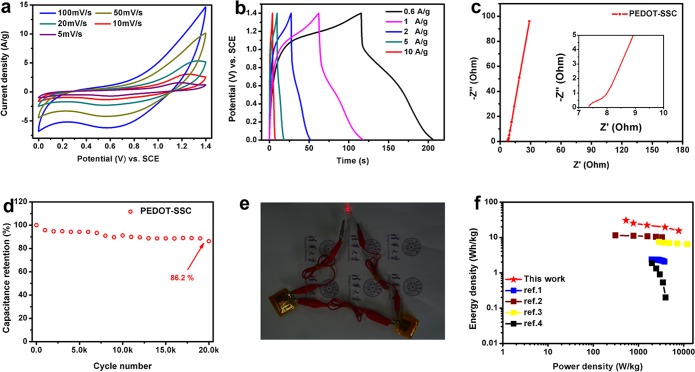
(a) CV curves of NNA@PEDOT-based symmetric supercapacitor (PEDOT-SSC) at different scan rates; (b) GCD curves of the PEDOT-SSC electrode at different current densities; (c) EIS of PEDOT-SSC (inset: magnified spectra in the high-frequency region); (d) cycling performance of PEDOT-SSC at 5 A/g; (e) two PEDOT-SSCs in series light up an LED; (f) Ragone plot of PEDOT-SSC compared to some of the most advanced energy-storage devices recently reported (based on the mass of the active materials).

A series of NNA@PEDOT samples were prepared with different mass loadings of 0.22, 0.75, 1.09, and 1.54 mg/cm^2^ as the vapor-phase polymerization cycles increased from 1 to 4, allowing the convenient control of the PEDOT loading on the NNA, as shown in Fig 5F. As expected, the current response increased with higher mass loadings, indicating improvements in the total capacitance (Figure E in S1 File). However, the specific mass capacitance decreased probably as a consequence of an increased film thickness and thus decreased effective polymer contribution to a specific capacitance value [32,35]. Fig 4B presents the CV curves of the NNA@PEDOT electrode at different scan rates with a fixed mass loading of 0.22 mg/cm^2^. According to Eq (1) in S1 File, the specific mass capacitance was 120.45 F/g and 211.98 F/g at 10 mV/s and 2 mV/s, respectively. The loss of capacitance at a higher scan rate could be attributed to the limited ion accessibility to the electrode [35]. In Fig 4D, the GCD curves of the NNA@PEDOT electrode at different current densities are displayed. Similar to the CV results, the specific capacitances of NNA@PEDOT were calculated to be 233.25 F/g and 85.00 F/g at 2 A/g and 10 A/g, respectively, according to Eq (2) in S1 File. The decreased capacitance at higher current density could be a consequence of limited ion diffusion [35].

In Fig 4E, the cycling performance of the NNA@PEDOT electrode was excellent during the whole experiment (95.7% capacitance retention after 20 000 cycles at 5 A/g), whereas that of the NF@PEDOT electrode decayed very quickly in the initial cycles and had a capacitance retention of only 59.5% after 20 000 cycles. The decreased capacity retention of NF@PEDOT could be associated with the structural pulverization as a result of repeated swelling and shrinking of the PEDOT chains [36], which is further demonstrated from the morphological evolution of NF@PEDOT and NNA@PEDOT before and after the cycling test in Figure F in S1 File. As clearly observed, the structure of NF@PEDOT severely cracked after the test. By contrast, these problems were alleviated, and the initial structural integrity was maintained with NNA@PEDOT. NNA@PEDOT showed an almost negligible activity loss after a long duration of testing, featuring the best cycle performance among the recently reported results for PEDOT-based electrodes [18,35,37–40]. This superior performance could be explained by the following reasons [23,27,28,32]: 1) the NNA skeleton provides continuous electron “highways” that could minimize the total resistance; 2) the super-hydrophilicity and open porous architecture help create fast channels for electrolyte ions throughout the framework; and 3) the proper surface convexity on the Ni nanowires can help release electrostatic stress during repeated doping and de-doping on the electrode/electrolyte interface. Moreover, compared with the electrochemical polymerization method, which is also commonly used, the vapor-phase polymerization method is more effective at preparing PEDOT with better regularity of the polymer chain structure, which is critical for improving the electrochemical properties of PEDOT. Moreover, compared with the electrochemical polymerization of EDOT, which requires a high working potential of approximately 1.2 V, vapor-phase polymerization requires a moderate working potential and thus can minimize unwanted side reactions.

NNA@PEDOT was also evaluated as the positive electrode in a potential window of 0 V~0.6 V (Figure G in S1 File) and showed good cycling performance of 84.3% retention after 20 000 cycles at 5 A/g.

To analyze the performance of NNA@PEDOT at the device level, we fabricated a symmetric supercapacitor composed of NNA@PEDOT//NNA@PEDOT (PEDOT-SSC) in an aqueous electrolyte (0.5 M Na_2_SO_4_) for further study. The PEDOT-SSC exhibited excellent performance in potential ranges of 0 V to 0.8 V and 0 V to 1.4 V with almost no polarization (Figure H in S1 File). Fig 5A and 5B shows the typical CV and GCD curves of the device, respectively. According to the GCD curve, the as-prepared PEDOT-SSC had a specific capacitance of 102.50 F/g at 1 A/g. The cycle stability test of PEDOT-SSC showed a capacitance retention of 86.2% after 20 000 cycles at 5 A/g. The above results are among the best performance parameters of PEDOT-based symmetric and asymmetric supercapacitors, as shown in Table 1 [35,37,39,40–43]. According to the EIS analysis in Fig 5C, the small series resistance of PEDOT-SSC (*Re* of 7.36 Ω and *Rct* of 0.88 Ω included) ensured fast charge transport between the electrode and electrolyte and may also have caused the well-defined cycling stability of the device. The maximum gravimetric energy density and power density were 30.38 Wh/kg and 529.49 W/kg (calculated according to Eq (3) and Eq (4) in S1 File), respectively, which are among the best reported results to date for PEDOT-based symmetric supercapacitors, as shown in the Ragone plot displayed in Fig 5F [18,37,38,44]. Additionally, the superior electrochemical performance of the device was further demonstrated by lighting a light emitting diode (LED, 1.8 V) (Fig 5E) with two fully charged PEDOT-SSC in series, demonstrating the potential of PEDOT-SSC in supercapacitor applications.

**Table 1:** Comparison of the major features and merits of NNA@PEDOT with previously reported PEDOT-based electrodes in terms of areal/specific capacitance and cycling performance.

materials	electrolytes	specific capacitance	cycling performance	year	ref
PEDOT nanotubes	1 M LiClO_4_	~140 F/g @ 50 mV/s	-	2008	[37]
graphene/PEDOT	1 M H_2_SO_4_	261 F/g @ 20 mV/s	93% after 10000 cycles	2013	[39]
graphene/PEDOT hydrogel	1 M Na_2_SO_4_	174.4 F/g @ 5 mV/s	-	2013	[40]
PEDOT film	ACN-Bu_4_NPF_6_	120 F/g @ 1 A/g	88.6% after 1000 cycles	2014	[35]
PEDOT/g-C_3_N_4_	1 M Na_2_SO_4_	200 F/g @ 2 A/g	96.5% after 1000 cycles	2015	[41]
graphene/PEDOT multilayer films	1 M H_2_SO_4_	154 F/g @ 300 mV/s	88% after 1000 cycles	2012	[42]
VPP PEDOT	1 M H_2_SO_4_	92 F/g @ 0.2 A/g	~70% after 600 cycles	2013	[43]
VPP PEDOT-SWCNTs	1 M H_2_SO_4_	137 F/g @ 0.2 A/g	~89% after 1000 cycles	2013	[43]
VPP PEDOT-RGO	1 M H_2_SO_4_	156 F/g @ 0.2 A/g	~90% after 1000 cycles	2013	[43]
NNA@PEDOT	0.5 M Na_2_SO_4_	191.25 F/g @ 5 A/g	95.7% after 20000 cycles	2016	this work

## Conclusion

In summary, we developed a facile vapor-phase polymerization deposition approach for the fabrication of an NNA@PEDOT core@shell structure as the electrode for aqueous SSC. The current challenging issues concerning PEDOT-based electrodes were critically addressed by combining the advantages of both high electronic and ionic transport characteristics of the NNA structure and the high quality of the PEDOT structure through the vapor-phase polymerization approach. This hierarchical nanostructured electrode delivered both high capacity (191.25 F/g at 5 A/g) and superior longevity (4.3% capacity loss after 20 000 cycles at 5 A/g). At the device level, PEDOT-SSC demonstrated a high energy density (30.38 Wh/kg at 529.49 W/kg) and excellent cycling performance (86.2% capacitance retention after 20 000 cycles at 5 A/g). We believe that this 3D nanometal@conducting polymer structure will contribute to the development of high-performance supercapacitors and help address the issues in other electrochemical energy storage systems.

## Supporting Information

S1 File Figure A. Elemental distribution investigation of NNA@PEDOT. Figure B. SEM images of NNA and NNA@PEDOT. Figure C. EIS plots of NNA@PEDOT and NF@PEDOT. Figure D. CV curves of bare NNA and NNA@PEDOT. Figure E. CV curves of NNA@PEDOT with different mass loading. Figure F. TEM and SEM images of different electrodes. Figure G. CV curves, GCD curves and cycling performance result of NNA@PEDOT. Figure H. CV curves of the PEDOT-SSC. Figure I. Electrical equivalent circuit used for fitting the impedance spectra.Calculations.
(DOCX)
