# Supplementary material for: Vapor-Phase Polymerized Poly(3,4-Ethylenedioxythiophene) on a Nickel Nanowire Array Film: Aqueous Symmetrical Pseudocapacitors with Superior Performance
Source: PLoS One. 2016 Nov 18;11(11):e0166529. doi: 10.1371/journal.pone.0166529 (PMC5115749; doi:10.1371/journal.pone.0166529)
Supplement: S1 File — Figure A. Elemental distribution investigation of NNA@PEDOT. Figure B. SEM images of NNA and NNA@PEDOT. Figure C. EIS plots of NNA@PEDOT and NF@PEDOT. Figure D. CV curves of bare NNA and NNA@PEDOT. Figure E. CV curves of NNA@PEDOT with different mass loading. Figure F. TEM and SEM images of different electrodes. Figure G. CV curves, GCD curves and cycling performance result of NNA@PEDOT. Figure H. CV curves of the PEDOT-SSC. Figure I. Electrical equivalent circuit used for fitting the impedance spectra.Calculations. (DOCX) [file pone.0166529.s001.docx]

TITLE:Vapor-Phase Polymerized Poly(3,4-ethylenedioxythiophene)

on a Nickel Nanowire Array Film: Aqueous Symmetrical

Pseudocapacitors with Superior Performance

Qisen Xie^1¶,^ Yang Xu^1¶^, Zhipeng Wang^1¶^, Chao Xu^1^, Peichao Zou^1^, Ziyin Lin^2^, Chenjie Xu^3^,

Cheng Yang^1*^, Feiyu Kang^1^, Ching-Ping Wong^2^

**Supporting Information**


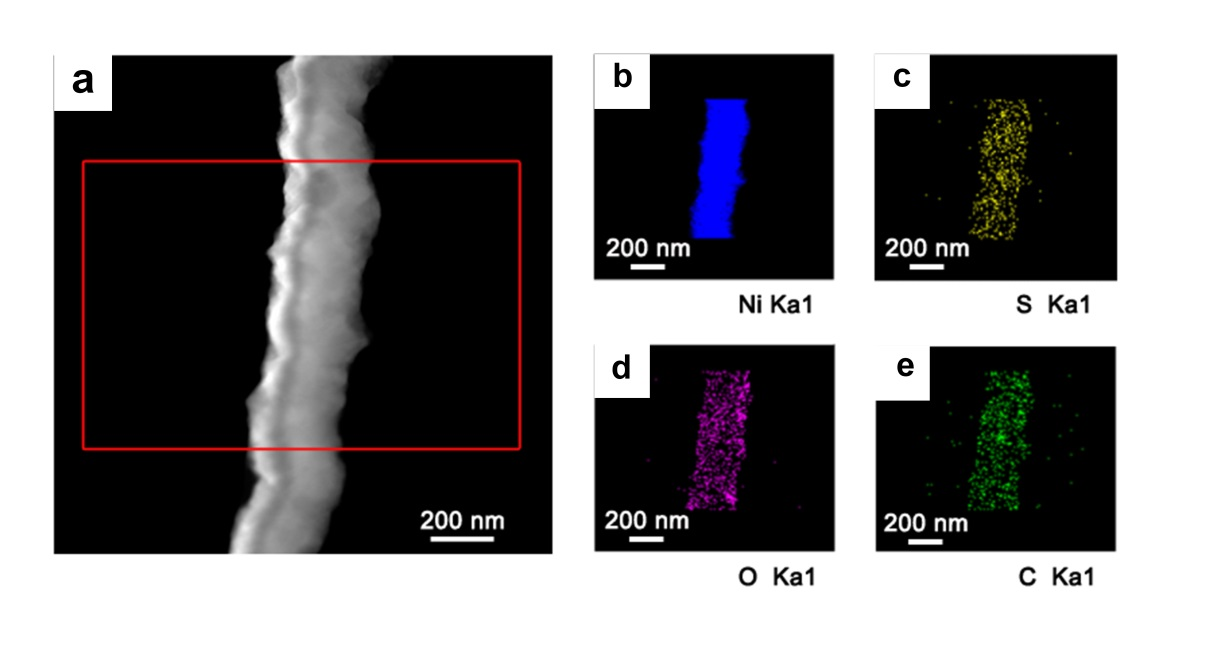


**Fig A. Elemental distribution investigation of NNA@PEDOT. (a) : TEM (dark field)**

**image of NNA@PEDOT sample. (b) to (e): element dispersive mapping images of Ni,**

**S, O, and C.**


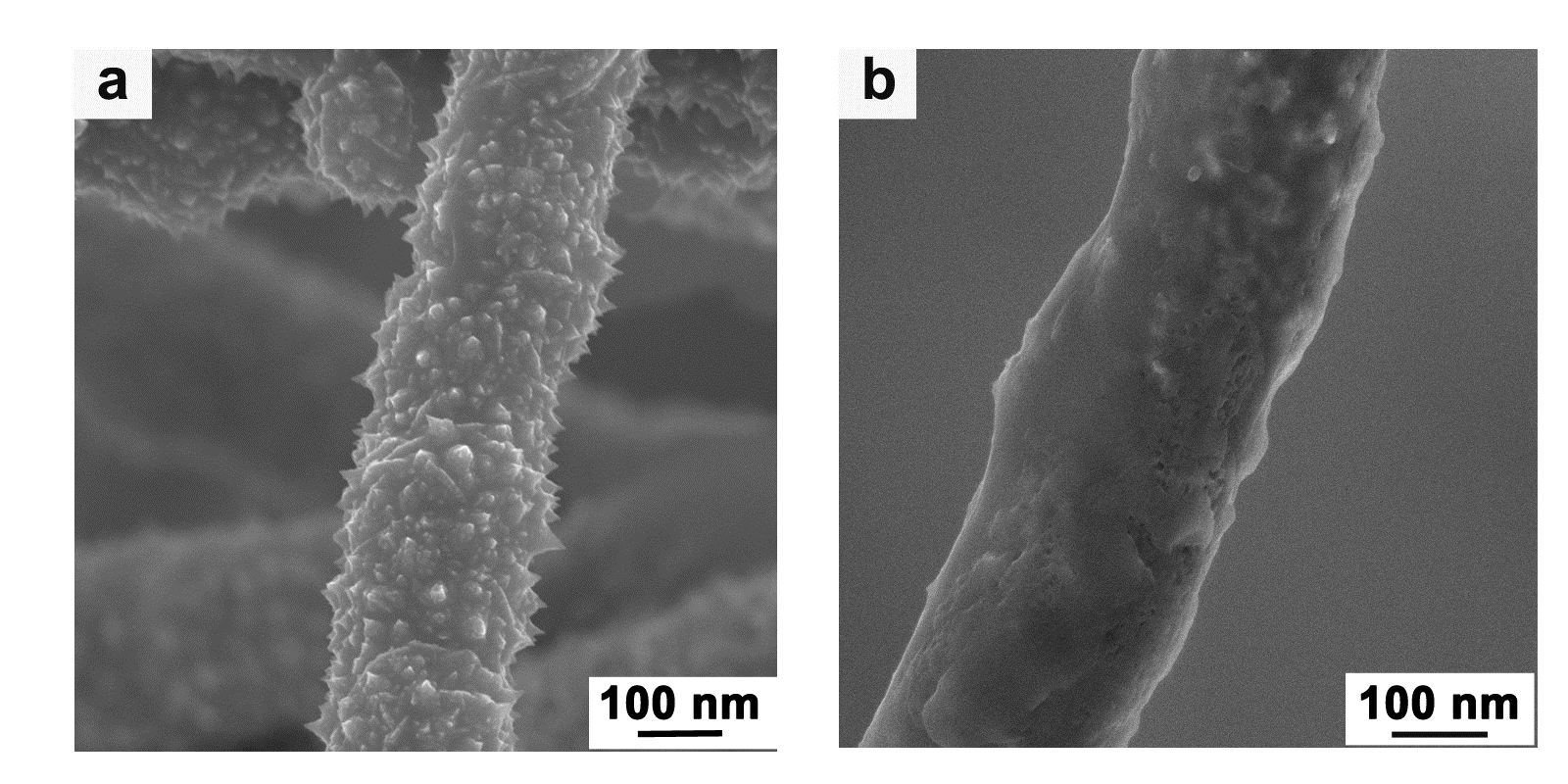


**Fig B. SEM images of (a) NNA and (b) NNA@PEDOT prepared via VPP.**


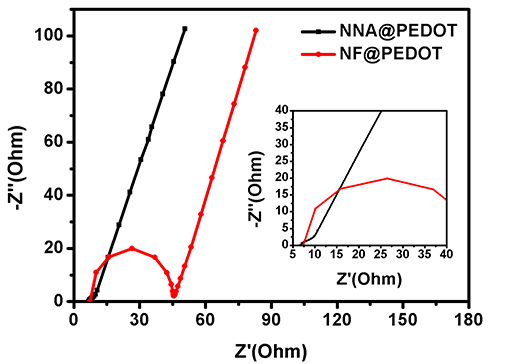


**Fig C. EIS plots of NNA@PEDOT and NF@PEDOT (inset: magnified plots in the**

**high-frequency region).**


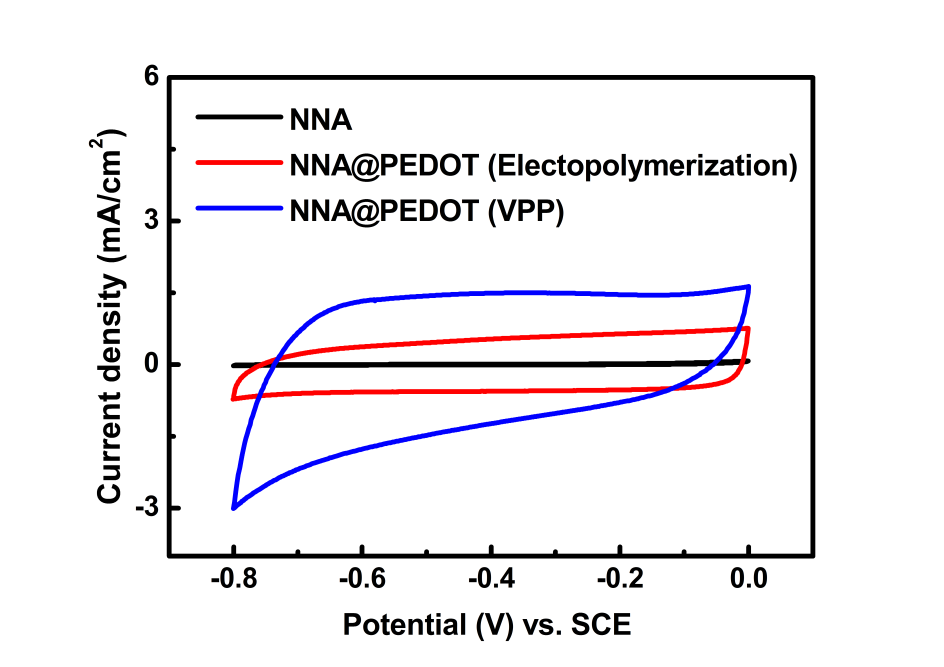


**Fig D. CV curves of bare NNA, NNA@PEDOT obtained from VPP and**

**electrochemical polymerization method.**


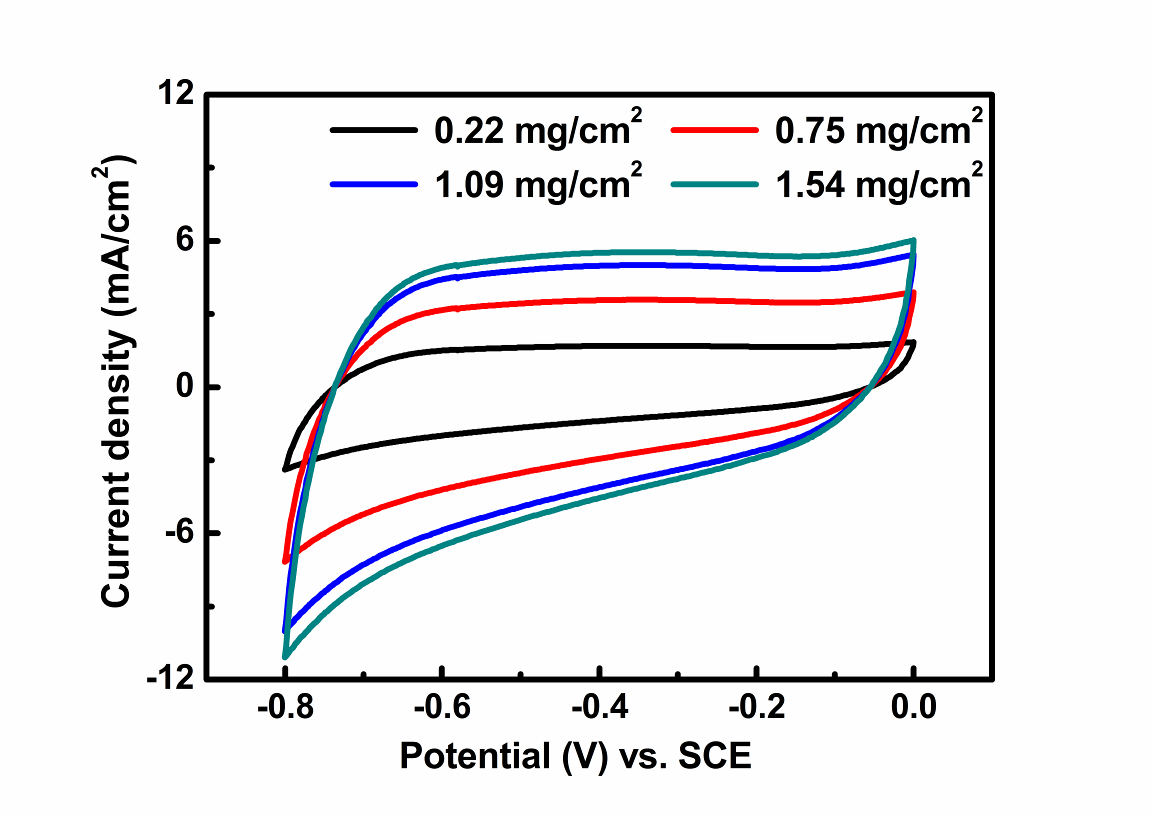


**Fig E. CV curves of NNA@PEDOT with different mass loading.**


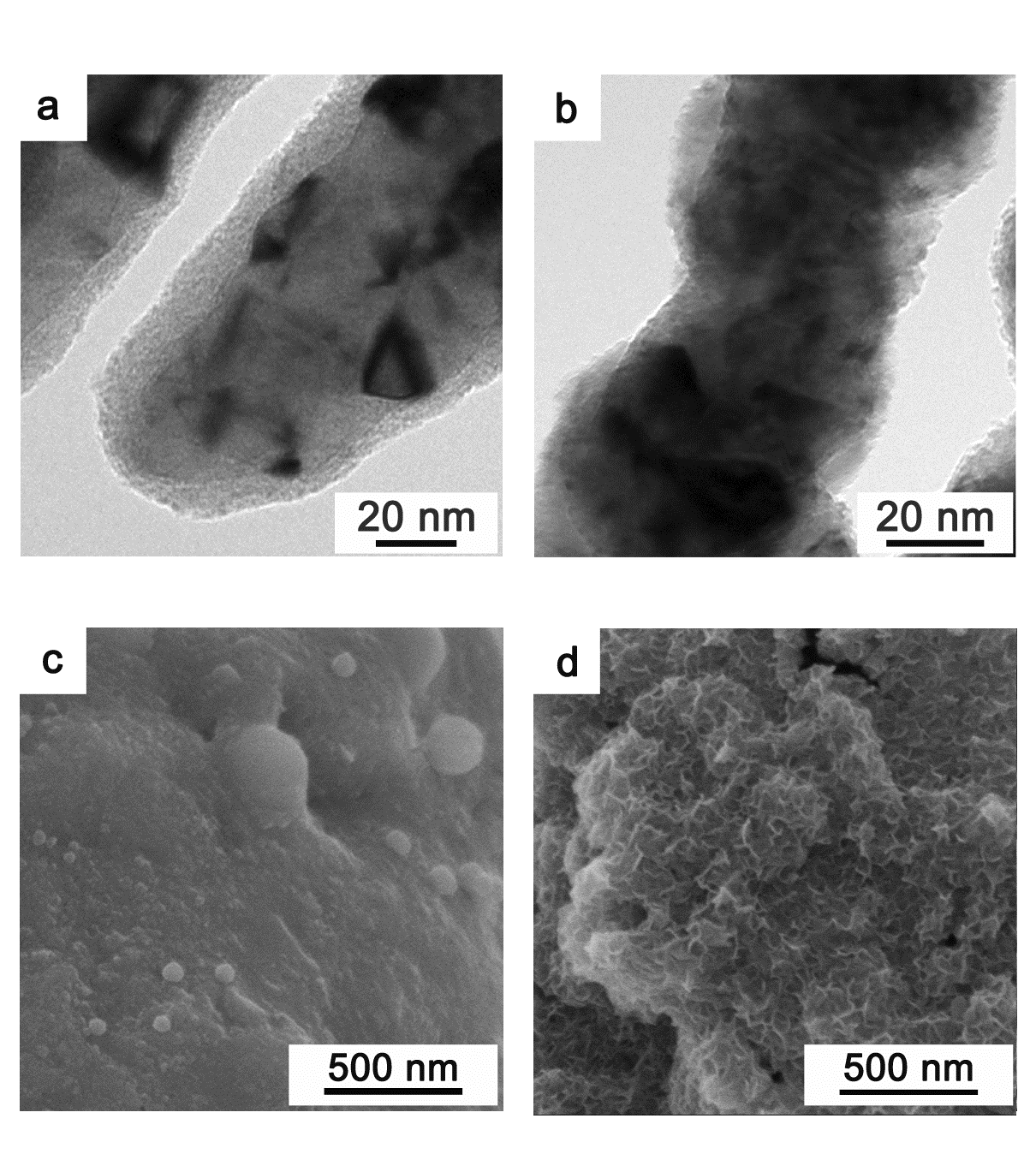


**Fig F. TEM and SEM images of the electrodes: (a) NNA@PEDOT before the cycle**

**test; (b) NNA@PEDOT after 20 000 cycles; (c) NF@PEDOT before the cycle test; and (d)**

**NF@PEDOT after 20 000 cycles.**


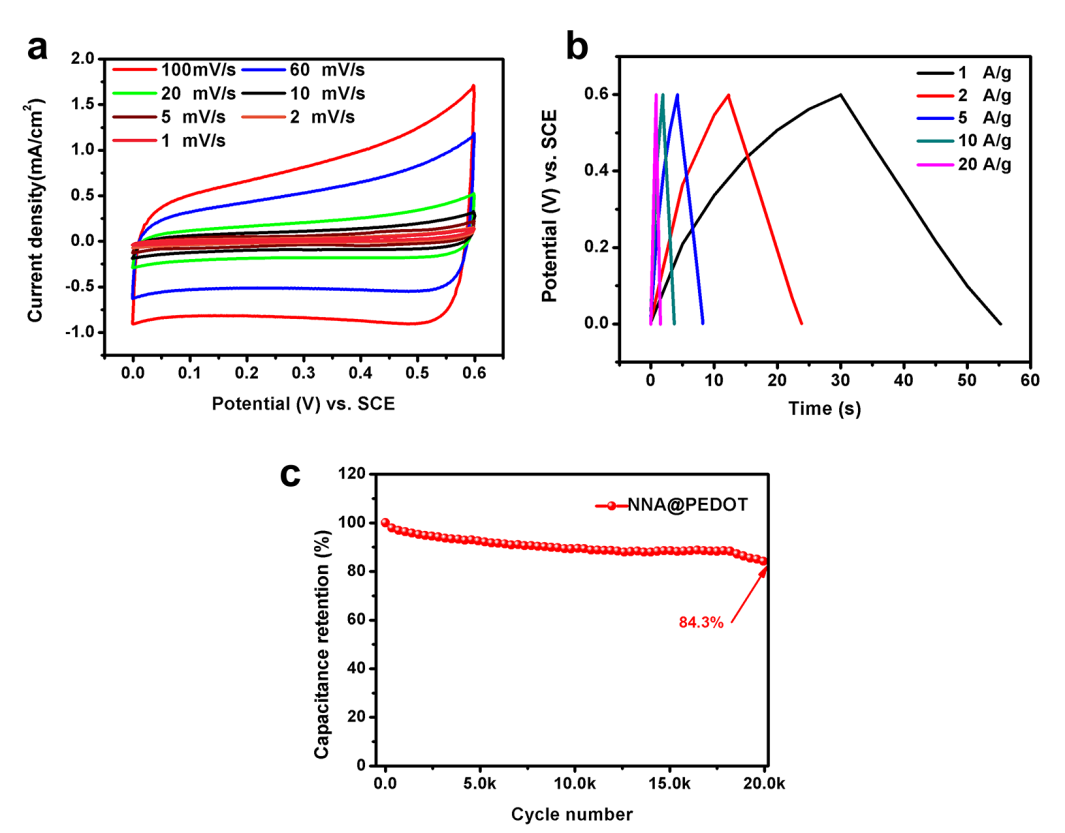


**Fig G. (a) CV curves of NNA@PEDOT in the potential window from 0 V to 0.6 V; (b)**

**GCD curves of NNA@PEDOT; (c) cycling performance result of NNA@PEDOT at 5 A /g.**


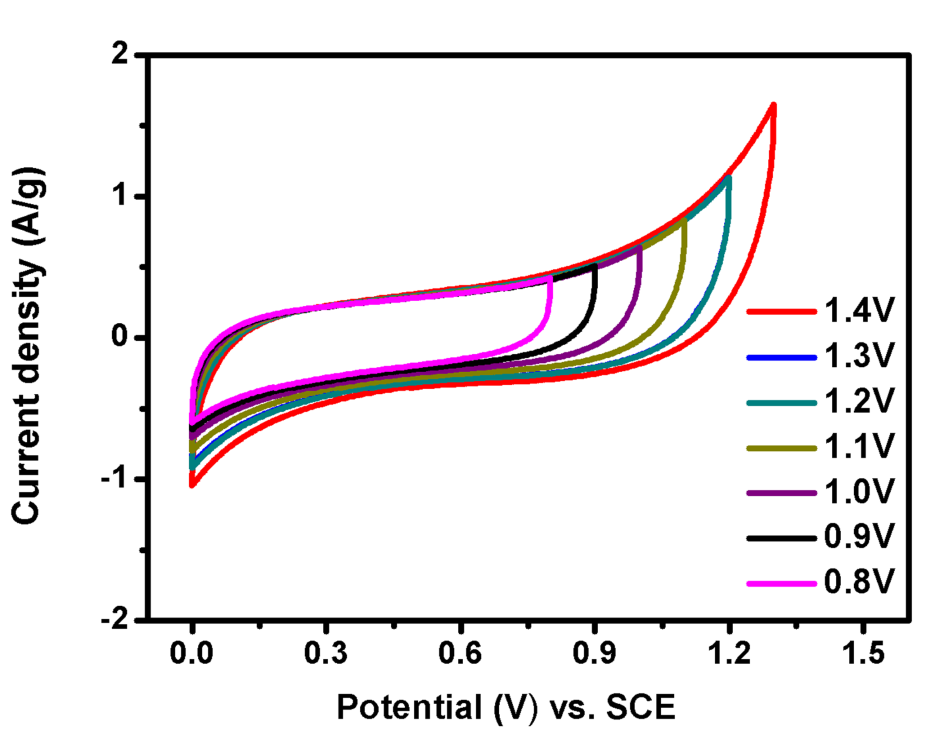


**Fig H. CV curves of the PEDOT-SSC with a potential range from 0 0.8 V to 0 1.4 V.**

**
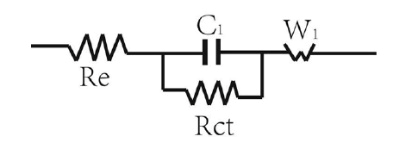
**

**Fig I. Electrical equivalent circuit used for fitting the impedance spectra.**

**Calculations.** The specific (mass or areal) capacitance can be calculated from the CV and

GCD curves according to the equations:

| $C=\frac{\int i\left（ V \right）dV}{m\cdot v\cdot\Delta V}$ | (1) |
| --- | --- |
| $C=\frac{I\cdot\Delta t}{m\cdot\Delta U}$ | (2) |

where C is the specific capacitance of the materials, *m* is the PEDOT mass loading density

on NNA@PEDOT electrode, *V* is the scan rate, $\Delta V$ is the potential window in the CV curves,

*i* (*V*) is the voltammetry current, *I* is the applied current, $\Delta U$ is the potential window in the

discharging process and $\Delta t$ is the discharging time. The energy and power density (*E* and *P*) are

calculated by the following equations:

| $E=\frac{\int IU\left（ t \right）d(t)}{m}$ | (3) |
| --- | --- |
| $P=\frac{E}{\Delta t}$ | (4) |

Where *I* is the charging current, *U* is the voltage, d*t* is the time differential, *t* is the

discharging time and *m* refers to the total mass of active materials of both positive and negative

electrode.

**References**

1. Liu R, Cho SI, Lee SB. Poly(3,4-ethylenedioxythiophene) nanotubes as electrode

materials for a high-powered supercapacitor. Nanotechnology. 2008; 19: 215710.

2. Sun D, Jin L, Chen Y, Zhang JR, Zhu JJ. Microwave-Assisted In Situ Synthesis of

Graphene/PEDOT Hybrid and Its Application in Supercapacitors. ChemPlusChem. 2013;

78: 227-234.

3. Zhou H, Yao W, Li G, Wang J, Lu Y. Graphene/poly(3,4-ethylenedioxythiophene)

hydrogel with excellent mechanical performance and high conductivity. Carbon. 2013;

59: 495-502.

4. Mo D, Zhou W, Ma X, Xu J, Zhu D, et al. Electrochemical synthesis and capacitance

properties of a novel poly(3,4-ethylenedioxythiophene bis-substituted bithiophene)

electrode material. Electrochimica Acta. 2014; 132: 67-74.

5. Chen X, Zhu X, Xiao Y, et al. PEDOT/g-C3N4 binary electrode material for

supercapacitors. journal of electroanalytical chemistry, 2015; 743:99-104.

6. Lee S, Cho M S, Lee H, et al. A facile synthetic route for well defined multilayer films of

graphene and PEDOT via an electrochemical method. Journal of materials chemistry,

2012; 22:1899-1903.

7. Yang Y, Zhang L, Li S, et al. Electrochemical performance of conducting polymer and its

nanocomposites prepared by chemical vapor phase polymerization method. Journal of

Materials Science: Materials in Electronics, 2013; 24:2245-2253.
